# Function and anatomy of plant siRNA pools derived from hairpin transgenes

**DOI:** 10.1186/1746-4811-3-13

**Published:** 2007-11-25

**Authors:** Bess L Chau, Kevin AW Lee

**Affiliations:** 1 Department of Botany, University of Hong Kong, Hong Kong S.A.R. China; 2 Department of Biology, Hong Kong University of Science & Technology, Hong Kong S.A.R. China

## Abstract

**Background:**

RNA interference results in specific gene silencing by small-interfering RNAs (siRNAs). Synthetic siRNAs provide a powerful tool for manipulating gene expression but high cost suggests that novel siRNA production methods are desirable. Strong evolutionary conservation of siRNA structure suggested that siRNAs will retain cross-species function and that transgenic plants expressing heterologous siRNAs might serve as useful siRNA bioreactors. Here we report a detailed evaluation of the above proposition and present evidence regarding structural features of siRNAs extracted from plants.

**Results:**

Testing the gene silencing capacity of plant-derived siRNAs in mammalian cells proved to be very challenging and required partial siRNA purification and design of a highly sensitive assay. Using the above assay we found that plant-derived siRNAs are ineffective for gene silencing in mammalian cells. Plant-derived siRNAs are almost exclusively double-stranded and most likely comprise a mixture of bona fide siRNAs and aberrant partially complementary duplexes. We also provide indirect evidence that plant-derived siRNAs may contain a hitherto undetected physiological modification, distinct from 3' terminal 2-O-methylation.

**Conclusion:**

siRNAs produced from plant hairpin transgenes and extracted from plants are ineffective for gene silencing in mammalian cells. Thus our findings establish that a previous claim that transgenic plants offer a cost-effective, scalable and sustainable source of siRNAs is unwarranted. Our results also indicate that the presence of aberrant siRNA duplexes and possibly a plant-specific siRNA modification, compromises the gene silencing capacity of plant-derived siRNAs in mammalian cells.

## Background

RNA interference (RNAi) culminates in gene silencing via the sequence specific action of small interfering RNAs (siRNAs). Originally described in plants [1,2] as post transcriptional gene silencing (PTGS), studies spanning the animal and plant kingdoms have uncovered a broad conservation of RNAi [3-6]. RNAi has been extensively reviewed [7-10] and the essential molecular mechanism involves processing of double-stranded (ds) precursor RNAs by a Dicer-like (DCL) endonuclease to produces 21–25 bp short interfering RNAs (siRNAs). Subsequently the siRNA/DCL complex is converted to an active RNA-induced silencing complex (RISC) containing the mRNA anti-sense siRNA strand (the guide) and an Argonaute (Ago) protein. In one of the major RNAi pathways (the RNA cleavage pathway) active RISC then performs a single endonucleolytic cleavage of the target RNA as directed by the guide siRNA.

RNAi has emerged as a powerful experimental tool and the size, stability and specificity of siRNAs points to significant commercial and therapeutic potential [11,12]. The discovery that synthetic siRNAs are competent for gene silencing [13,14] has proven particularly useful in mammalian cells by circumventing the toxic effects elicited by longer dsRNAs [15]. However while synthetic siRNAs are readily obtained their use is impacted by high costs and the variable potency of individual siRNAs. Use of recombinant Dicer to generate siRNA pools (Dsi) from larger dsRNA precursors in vitro [16,17] overcomes the latter problem but is still costly. It therefore remains of significance to seek more economical, scalable and sustainable methods for siRNA production. Exploitation of transgenic organisms for production of biomolecules is increasingly common and in this study we aimed to explore the potential of plants for heterologous siRNA production.

Besides small differences in size and their participation in a wide range of siRNA functions [18-20], broadly conserved siRNA structure suggests that siRNAs *per se *are likely to be functionally equivalent. Thus siRNAs might be expected to retain cross-species function but, for technical reasons, this issue has yet to be rigorously explored. One study [21] directly tested plant siRNAs in *C. elegans *and concluded that gene silencing activity co-purifies with a putative RNA species of approximately 80 nt. However, as noted by the authors [21] the low concentration of plant siRNAs present did not allow a conclusion concerning siRNA functionality in the worm. Another consideration is that due to asymmetric incorporation of double-stranded siRNAs into RISC in vivo [10,22], siRNAs extracted from plants might be single-stranded (as is the case for microRNAs [23-25]) and hence much less active [26]. Indirect cloning approaches [27] have provided some evidence that plant siRNAs from hairpin transgenes contain much single stranded siRNA but the siRNAs have not been directly examined. Finally, although a recent study concluded that plant-derived siRNAs are functional for gene silencing in mammalian cells [28] the efficiency of gene silencing was not established.

Here we report that siRNAs produced from plant hairpin transgenes and extracted from plants are ineffective for gene silencing in mammalian cells. Scalable and sustainable production of siRNAs cannot therefore be achieved using the above approach. Our results also indicate that the presence of aberrant siRNA duplexes and possibly a plant-specific siRNA modification, compromises the silencing capacity of plant-derived siRNAs in mammalian cells.

## Results

### Expression of mouse caspase 9 siRNAs in transgenic plants

**Figure 1:**
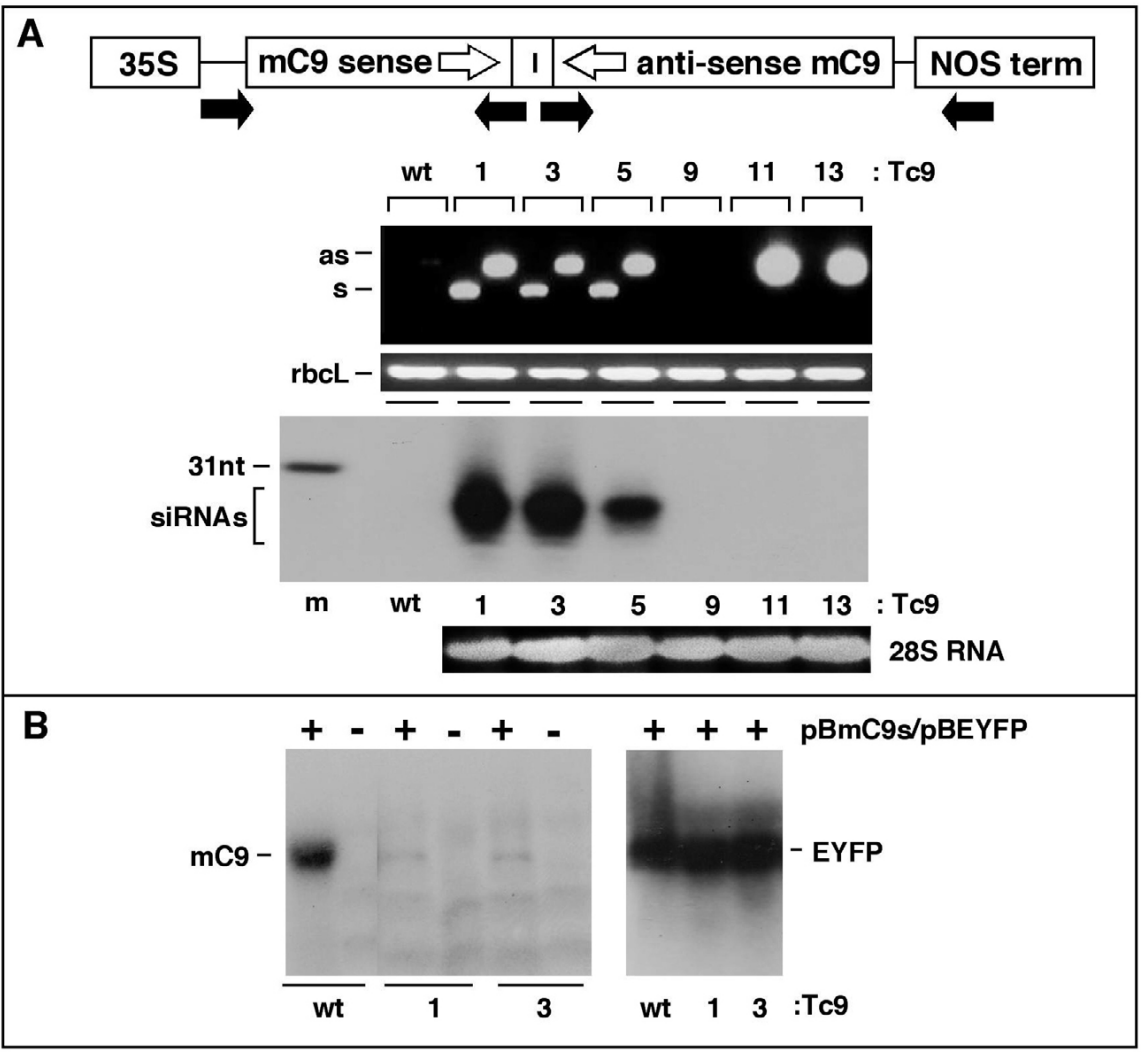
**Generation of transgenic tobacco expressing mC9 siRNAs**. **(A) **mC9 transgene and siRNA expression. The mC9 siRNA cassette contains 400 bp mC9 sense and anti-sense sequences separated by the TGA1 intron (I) with expression by the CMV 35S promoter (35S) and NOS terminator. Transgene integrity for plants Tc9 #1, 3, 5, 9, 11, 13 was verified via PCR (primer position indicated by black arrows) to detect sense (s) and anti-sense (as) transgene elements. Endogenous rbcL is control. For siRNA expression 30 μg of total RNA from wt tobacco or Tc9 transformants was analysed by Northern blotting. A 31 nt mC9 DNA oligo (m) is size marker and an overnight phosphor-image is shown. **(B) **In vivo gene silencing in Tc9 transformants. Transient transformation was performed by co-infiltration of tobacco wt, Tc9 #1 and #3 with plasmids expressing mC9 sense (pBmC9s) and EYFP transcripts (pBEYFP). Transcript levels in leaf regions were analysed 48 hr post-infiltration by Northern blotting (as described in materials and methods).

We aimed to evaluate whether siRNAs targeting a mammalian gene (mouse caspase 9, mc9) can be efficiently produced in transgenic plants and whether such siRNAs are competent for gene silencing in mammalian cells. Transgenic tobacco plants were obtained as previously described [28,29] using pBmC9 (Figure 1A). The expression cassette contained ~400 nt of caspase 9 sequence in the sense orientation with the corresponding antisense sequence downstream, separated by a natural intron that is known to facilitate siRNA expression [28]. The primary transcript is a 400 bp caspase 9 dsRNA stem that is recognised by the plant PTGS machinery and processed to yield a pool of siRNAs. Multiple kanamycin resistant/GUS positive plants (referred to as Tc9 #1, 3, 5, 9, 11 and 13) were examined for integration of the siRNA expression cassette by PCR of genomic DNA (Figure 1A). Transformants Tc9 #1, 3 and 5 contained an intact transgene while Tc9 #11 and 13 had only the anti-sense region and Tc9 #9 had possibly no transgene insertion at all. The above transformants were screened for siRNA expression by Northern blotting using a caspase 9 riboprobe (Figure 1A). Three of six transformants (1, 3 and 5) expressed readily detectable levels of caspase 9 RNA species that migrated slightly faster than a 31 nt oligonucleotide marker. The other transformants (9, 11 and 13) were negative (as was parental (wt) tobacco) as predicted by the lack of an intact transgene. Thus we conclude that Tc9 #1, 3 and 5 express the caspase 9 dsRNA precursor and efficiently produce a pool of siRNAs (referred to as Tc9si).

To verify the functionality of Tc9si we co-infiltrated transgenic plants with expression vectors for caspase 9 and EYFP RNAs (as control), isolated total plant RNA and probed for caspase 9 and EYFP transcripts by Northern blotting (Figure 1B). Caspase 9 transcripts were readily detected in wt infiltrated plants but only very low levels were detected in Tc9 Lines 1 and 3. EYFP transcripts were detected at similar levels in both wt and Tc9 plants. From the above results we conclude that Tc9 #1 and 3 express functional siRNAs directed against caspase 9 and since Tc9 #1 shows the highest Tc9si level (Figure 1A) we used this for Tc9si production.

### Analysis of Tc9si by RNAse protection assay

**Figure 2:**
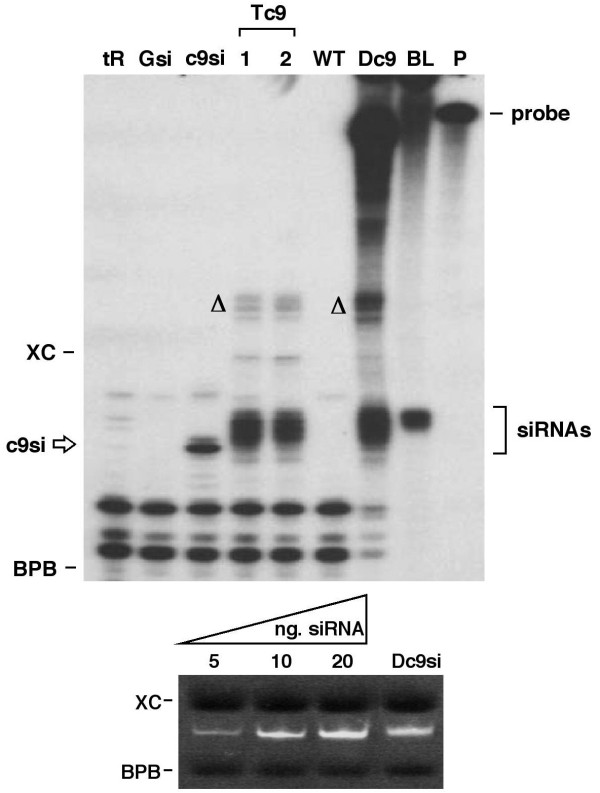
**siRNA detection by RNase Protection Assay (RPA)**. Synthetic mC9 siRNA (c9si) and different sources of RNA were probed by RPA (top) using a mouse caspase 9 anti-sense probe. The positions of intact ^32^P-labeled probe (~200 nt), siRNAs, xylene cyanol (XC, ~60 bp) and bromophenol blue (BPB, ~15 bp) are indicated. tR (yeast tRNA); Gsi (GAPDH synthetic siRNA); c9si (single mC9 synthetic siRNA); Tc9 1 and 2 (total RNA samples from two Tc9 plants); WT (total RNA from wt plant); Dc9 (Dicer-derived mC9 siRNA); BL (body-labeled Dicer siRNA). Bands marked Δ are artifacts of the RPA as described in the text. Quantitation of Dc9si (bottom) was achieved by comparison with known amounts of synthetic siRNAs following resolution on 15% non-denaturing PAGE gels and staining with EtBr.

Although Tc9si can be detected by Northern blotting the signal tended to be quite variable and with low resolution. We therefore employed a highly sensitive RNAse protection assay (RPA) and initially used a 21 nt caspase 9 siRNA (c9si) to establish conditions. An ~200 nt ^32^P-labeled caspase 9 riboprobe was hybridised with denatured c9si, hybrids were treated with a limited amount of RNAses A/T1 and protected fragments were resolved on a denaturing polyacrylamide gel (Figure 2). A single c9si-dependent band with a mobility consistent with ~21 nt was observed with two smaller species that result from interaction of the probe with carrier tRNA. Using RPA it is quite feasible to detect as little as 10 pg of c9si, as reported by others [30,31].

Because Tc9si contain many individual siRNAs each with different efficacy it is not appropriate (for the gene silencing assays described later) to compare Tc9si with a single synthetic siRNA such as c9si. As a positive control we therefore used human recombinant Dicer [16,17] to produce siRNA pools (Dc9si) in vitro from the same caspase 9 target region used to create Tc9 plants. Dicer was used to produce Dc9si followed by RPA analysis. The Dc9si sample yielded several products in the size range of 22–25 nt (Figure 2) as expected and similar to but broader than Dc9si that were directly body-labeled (BL) using radioactive UTP in the in vitro transcription reaction. Dc9si also produced some additional slower migrating species (marked Δ in the figure) but these are absent in BL and therefore appear to be an artifact of the RPA. Dc9si were quantified by comparison with known amounts of synthetic siRNAs after ethidium bromide staining (Figure 2).

Analysis of total RNA samples from Tc9 plants showed a pattern of protected fragments very similar or identical to that obtained for Dc9si and that were absent in RNA samples from parental (wt) plants (Figure 2). The RPA therefore faithfully detects Tc9si present in total plant RNA samples. In addition the RPA assay readily allows detection of larger dsRNA precursors but for several tobacco RNA samples (including those shown in Figure 2) none were detected. Thus dsRNA processing from transgenes appears to be highly efficient in tobacco and allows for direct testing of total plant RNA in mammalian cells without concern for PKR activation that would be brought about by longer unprocessed dsRNAs [15].

### Efficiency of plant siRNA production

We employed TRIzol reagent (Invitrogen) for plant RNA extraction and optimised the amount of TRIzol required (see materials and methods). Optimised TRIzol extraction gave ~50 ng of Tc9si/340 μg of total RNA/g of tobacco leaf (as determined by RPA of Tc9si and known amounts of Dc9si) equating to ~1.5% by mass compared with total plant mRNA. Similar siRNA levels were produced by transgenic tomato fruit (data not shown) and agree with expectations based on CaMV 35S-driven protein expression plants [32]. We also calculate that the cost of plant siRNA production is broadly comparable with that for in vitro-generated siRNA pools (produced by recombinant Dicer) but much greater than that for single synthetic siRNAs (data not shown).

### A rapid small scale silencing assay for Tc9si

**Figure 3:**
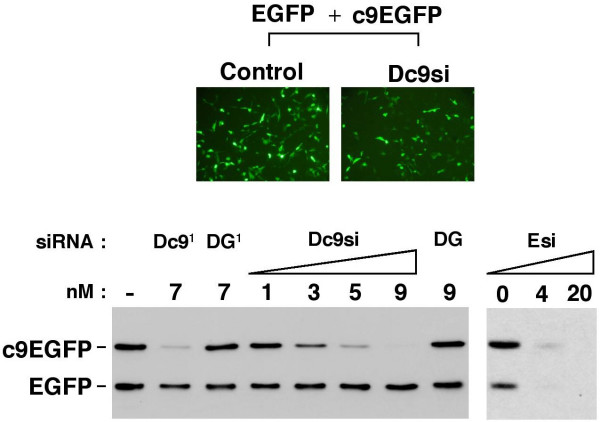
**Mammalian cell gene silencing assay**. Mouse BalbC 3T3 cells in a 24-well tissue culture plate format were transfected with equal amounts (500 ng each) of expression vectors for EGFP (pdsEGFP) and a mouse caspase 9/EGFP fusion (pmC9-dsEGFP) in the presence of the indicated concentrations (nM) of siRNAs. The fluorescent image (top) shows EGFP/c9EGFP expression in transfected cells. siRNAs used are as follows: Dc9si (gel purified Dicer-generated caspase 9); DGsi (gel purified Dicer-generated GAPDH); Esi (synthetic EGFP siRNA). DGsi/DGsi^1 ^and Dc9si/Dc9si^1 ^refer to two different siRNA preparations. Silencing was assessed at 16 hours post-transfection by Western blotting using EGFP antibody and 15% of the protein extract from a single well of the tissue culture plate (as described in materials and methods).

Given the yield of Tc9si we anticipated that a small scale mammalian gene silencing assay would be required for functional testing. We found that Lipofectamine2000 (Clontech) mediated co-transfection of BalbC-3T3 cells with an EGFP siRNA (Esi) and expression vectors for EGFP and a caspase 9-EGFP fusion protein (c9EGFP) (Figure 3) enabled sensitive monitoring of silencing via Western blotting with EGFP antibody (JL8, Clontech). Under the conditions tested Esi was highly effective (at 4 nM) at silencing both EGFP and the c9EGFP fusion.

Before testing Tc9si we first established conditions for gene silencing by gel-purified Dc9si (Figure 3). Addition of gel purified Dc9si during transfection efficiently reduced c9EGFP protein levels without any effect on EGFP, while the same amount of an unrelated Dicer-generated GAPDH siRNA (DGsi) had no effect. 9 nM Dc9si was sufficient to almost eliminate c9EGFP expression, while 5 nM (corresponding to 3 ng of Dc9si per well) was very effective and 3 nM had a significant although weaker effect. We conclude that Dc9si are very effective at silencing the c9EGFP target under the assay conditions employed and that silencing can readily be monitored using 5 nM Dc9si.

**Figure 4:**
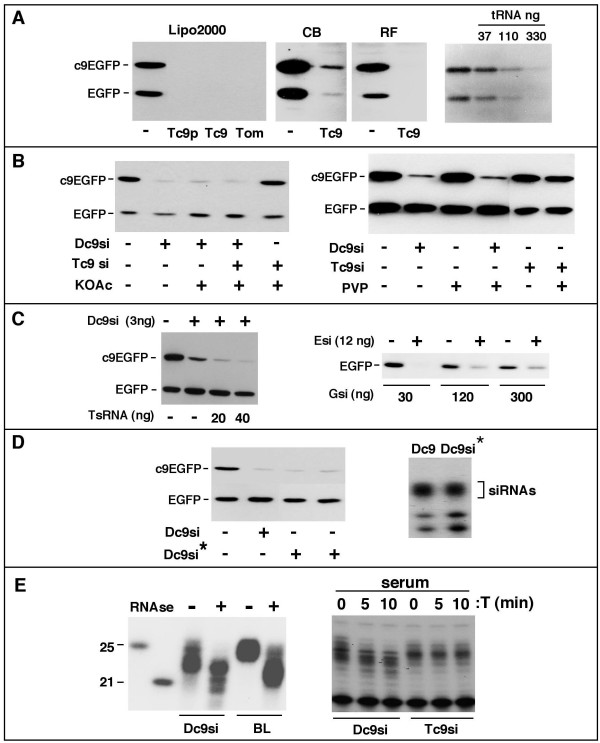
**Testing plant-derived siRNAs in mammalian cells**. **(A) **Testing plant siRNAs for gene silencing. Gene silencing assays were performed in 96 well plate format. Cells were co-transfected with pmC9-dsEGFP and pdsEGFP (70 ng each) together with 18 μg of total Tc9 RNA (Tc9), 10 μg of total tomato RNA (Tom), gel purified Tc9 small RNAs (Tc9p) contributing 5 nM Tc9si or tRNA (amount indicated in ng) using Lipofectamine 2000 (Lipo2000), Code Breaker (CB) or RNAiFect (RF) transfection reagents. **(B) **Effect of KOAc and PVP treatment. Left Hand Side. Silencing assays contained 3 ng of gel-purified Dc9si and Tc9 small RNAs containing 3 ng of Tc9si that had been pre-treated (or not) with 200 mM KOAc (see materials and methods). Right Hand Side. Assays were performed with 3 ng of KOAc treated siRNA samples in the presence and absence of 2% Polyvinylpyrrolidone (PVP) (see materials and methods). **(C) **Effect of other small RNAs on gene silencing. Left Hand Side. Silencing assays were performed with increasing amounts (indicated in ng) of gel-purified small RNAs (TsRNAs) from wt tobacco plants together with Dc9si. Right Hand Side. Cells were co-transfected with pdsEGFP only and one amount of Esi (12 ng) together with increasing amounts of a synthetic GAPDH siRNA (Gsi). **(D) **Effect of plant small RNA preparation protocol on Dc9si activity. Dc9si was mixed into the tobacco wt plant sample (at 50 ng/g of leaf) during the RNA extraction process. Extracted RNAs were gel purified and recovered Dc9si (Dc9si^*^) was quantitated by RPA (right panel). For silencing assays, cells were co-transfected with pmC9-dsEGFP and pdsEGFP with 3 ng of Dc9si or 3 ng of two different preparations of Dc9si^*^. **(E) **Effect of RNase digestion and serum treatment on siRNAs. Left hand side. Unlabeled Dc9si and ^32^P-body-labeled Dc9si (BL) were treated (treated (+) or untreated (-)) with a limited amount of RNAses as described in materials and methods. Dc9si was detected by RPA and BL Dc9si was detected directly on the same gel. ^32^P-end labeled synthetic RNA oligonucleotides of 25 and 21 nt are size markers. For serum treatment (right hand side), Dc9si and Tc9si were treated with FBS (see materials and methods) for the indicated times. FBS treated products were detected by RPA.

In light of the relatively low abundance of Tc9si in total plant RNA samples and several other technical challenges (see Figure 4) the small scale, rapidity and robustness of the above assay greatly facilitated experiments with plant materials that were cubersome to produce. In addition early microscopic monitoring of EGFP expression from the control vector just a few hours post-transfection (Figure 3) was expeditious for problem solving during trial experiments.

### Tc9si activity in mammalian cells

Using the above assay we attempted to test Tc9si for silencing activity (Figure 4) but found that Tc9 total RNA (or total RNA from tomato, data not shown) almost eliminated expression of c9EGFP and EGFP (Figure 4A). The same effect was observed using other cells (HeLa, data not shown) and other transfection reagents including Codebreaker and RNAifect (Figure 4A) and there was no effect of Tc9 RNA on endogenous β-actin levels (data not shown). As little as 100 ng of yeast tRNA mimicked the inhibitory effect of the Tc9 RNA sample indicating that plant RNA is most likely responsible for the inhibition.

To try and overcome the above problem we gel purified Tc9si (Tc9p) as described for Dc9si but this did not remove the inhibitory effect (Figure 4A). Since plant RNA preparations are often contaminated with polysaccharides and polyphenolic compounds we next treated purified Tc9si sample with 200 mM KOAc to remove putative contaminating polysaccharides [33] and found that this eliminated the inhibitory effect (Figure 4B, left hand panel). Surprisingly however the Tc9si sample had no silencing activity despite containing easily sufficient c9siRNA as assessed by RPA. KOAc does not reduce silencing by Dc9si and, most importantly, the Tc9si sample has no inhibitory effect on silencing by Dc9si. Polyvinylpyrrolidone (PVP) has been shown to overcome the inhibitory effect of polyphenolic compounds on certain reactions, such as rtPCR, involving plant RNA [34]. We therefore treated the Tc9 sample with PVP but found that this did not lead to activation of the Tc9si sample (Figure 4B). We also tested up to 10 nM Tc9si (for technical reasons we could not assay higher concentrations) and observed no silencing activity (data not shown). The above data indicate a substantial difference in the activity of Dc9si (3 nM is active and 10 nM is very potent, Figure 3) versus Tc9si (10 nM is not active).

The gel purified Tc9si sample contains a significant amount of endogenous small RNAs, including microRNAs [35,36] that might interfere with silencing by competing with Tc9si for a limiting amount of RISC [37]. To examine the above possibility we first prepared gel-purified plant small RNAs (Total small RNAs or TsRNAs) from wt plants and assessed the amount of contaminating TsRNAs present in the Tc9si sample. Quantitation of TsRNAs by comparison with known amounts of siRNA by ethidium bromide staining (as described for quantitation of Dc9si) and estimation of Tc9si by RPA, showed that there is a ~10:1 ratio of TsRNAs : Tc9si and thus the silencing assays contain ~30 ng of TsRNAs and 3 ng of Tc9si. However, testing of TsRNA up to 40 ng in the silencing assay does not inhibit silencing by 3 ng of Dc9si (Figure 4C). To further evaluate the presence of putative si/miRNA inhibitors in the Tc9si sample we tested the effect of a synthetic GAPDH siRNA (Gsi) on silencing by EGFP siRNA (Esi). Up to 30 ng of Gsi has no effect on EGFP silencing while 300 ng of Gsi still allows some silencing (Figure 4C). The above competition analyses demonstrate that lack of silencing by Tc9si is not due to inhibition by a contaminating component within Tc9si that competes for limiting RISC.

Although competent for nucleic acid hybridisation (as shown by RPA) it remained possible that Tc9si may be chemically damaged during preparation in a manner that impairs gene silencing. To test this we mixed Dc9si with a plant RNA extract at the first step of RNA preparation, recovered and quantified the treated Dc9si (referred to as Dc9si^*^, Figure 4D) and tested for silencing activity. Dc9si_^*^_retained full silencing activity and we therefore conclude that the procedure for Tc9si isolation does not inactive Tc9si.

Finally because siRNAs can be degraded by serum nucleases [38] we tested the effect of serum treatment on the Dc9si and Tc9si samples (Figure 4E) to verify that the inactivity of Tc9si is not due to instability in tissue culture. Serum treatment of Dc9si produced a gel mobility shift on denaturing gels similar to that observed for digestion with low amounts of RNAseA/T1 and consistent with a limited "exonucleolytic" cleavage (as described below) while Tc9si present in the gel purified sample is actually resistant to limited digestion by serum nucleases (Figure 4E). We conclude that lack of gene silencing by Tc9si is not due to instability of Tc9si in cell culture. From all of the above control experiments we conclude that siRNAs extracted from plants are insufficient for gene silencing in mammalian cells.

### siRNAs extracted from plants are in duplex form

**Figure 5:**
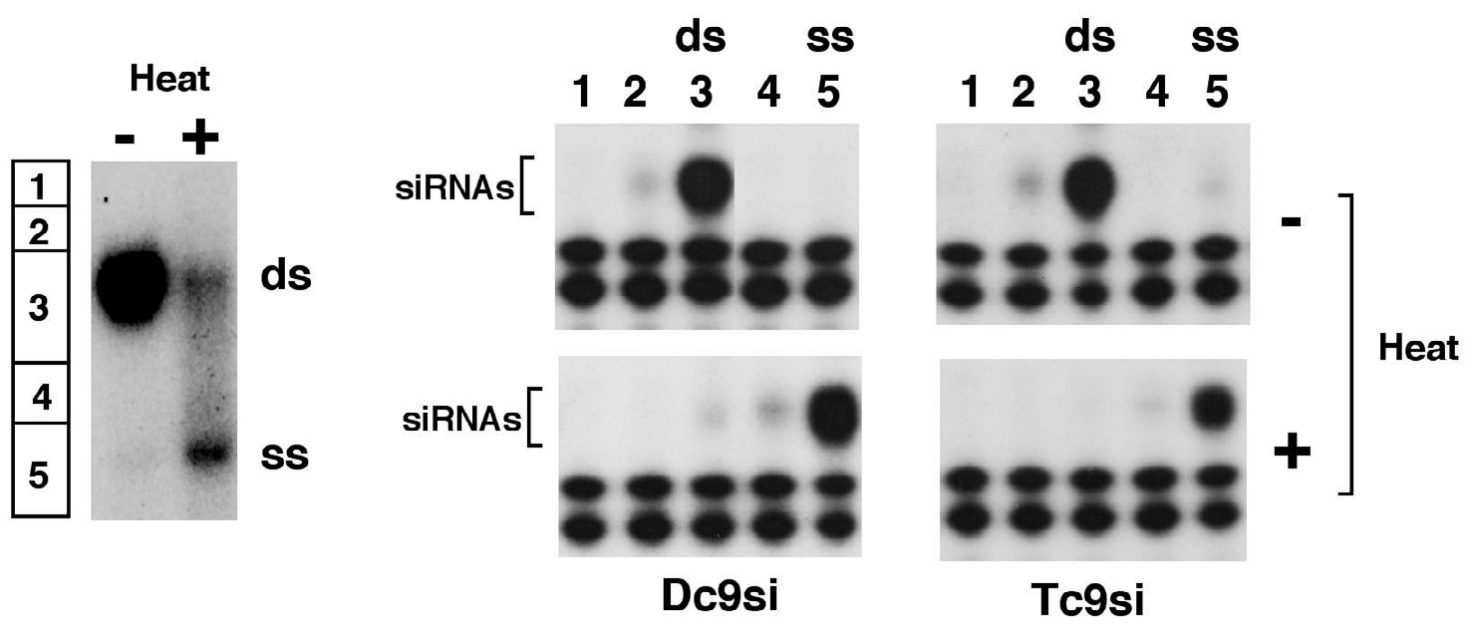
**Detection of double- and single-stranded siRNAs**. 32P-end labeled double-stranded siRNA (-) was heat denatured (+) and run on a non-denaturing polyacrylamide gel to provide mobility markers for ds and ss siRNAs (left hand side). Gel-purified Tc9si and Dc9si were heat denatured and separated on a non-denaturing gel along with the mobility markers described above. Regions of the gel marked 1–5 (left hand side) and containing Tc9si and Dc9si were excised, RNA recovered and detected by ribonuclease protection assay on denaturing gels (right hand side). Gel slice number is indicated at the top and heat treatment to the right.

In the asymmetric RISC formation process in vivo [10,22] only one strand of each siRNA is retained in active RISC while the other strand is degraded. This suggested that siRNAs extracted from plants might be predominantly single stranded thus explaining their lack of gene silencing activity [26] and we therefore determined the proportion of single-stranded siRNAs in Tc9si and Dc9si. siRNAs were heat-denatured and resolved on a non-denaturing polyacrylamide gel together with a ^32^P-labeled synthetic siRNAs to provide a marker for single and double-stranded siRNAs (Figure 5). RNAs were eluted from different regions of the gel and detected by RPA under denaturing conditions. The results show that Tc9si and Dc9si samples are very similar and that the majority of Tc9si are double-stranded with barely detectable levels of single-stranded siRNA (Figure 5). Thus lack of gene silencing by Tc9si in mammalian cells is not due to the presence of high amounts of single-stranded Tc9si RNAs. The extent to which the double-stranded RNAs present in the Tc9si sample represent bona fide siRNAs is, however, not established by our experiment (see discussion).

### Effect of siRNA 3'-2-O-methylation on mammalian gene silencing

**Figure 6:**
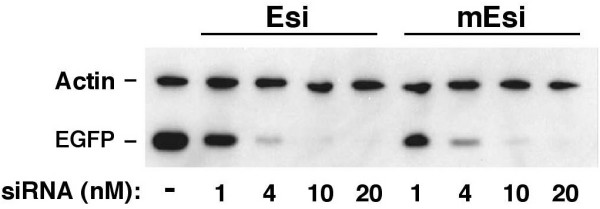
**Effect of siRNA methylation on gene silencing**. EGFP (Esi) and 3'-2-O methylated EGFP (mEsi) siRNAs were tested for gene silencing using a co-transfected EGFP target and the indicated concentrations of siRNAs. Endogenous β-Actin served as negative control.

All classes of *Arabidopsis *[39] and tobacco [40] siRNAs appear to have 3' terminal 2-O methylations and many studies have tested the silencing activity of methylated siRNAs in mammalian cells [38,41-43]. In one case [43] a single 3' 2-O methylation had no effect on silencing although this was under high siRNA concentration (100 nM) which might mask sub-optimal activity [37,44]. We therefore tested a synthetic 3' 2-O methylated EGFPsiRNA (mEsi) and the corresponding unmethylated control (Esi) under stringent assay conditions (4 nM siRNA) and found no obvious effect of methylation on the efficiency of silencing (Figure 6). Thus 3' 2-O methylation of siRNAs in planta does not appear to explain the lack of silencing activity observed for Tc9si in mammalian cells.

### Putative physiological modification of Tc9si

In light of the above result for methylated siRNAs we probed for evidence of additional structural modification of Tc9si. RPA analysis (Figure 2) showed no obvious size difference between Dc9si and Tc9si but to better calibrate the size range we ran ^32^P-labeled 21 nt and 25 nt siRNA size markers and treated Dc9si with limiting amounts of RNase A and T1 (Figure 4E). This produced a discreet increase in mobility of 2–3 nt for Dc9si, consistent with selective digestion of single stranded siRNA overhangs as previously described [30]. Thus limited digestion of siRNAs with endonucleases (such as RNAseA/T1 or those reportedly present in serum [38]) appears to be exonucleolytic due to the resistant double stranded body of the siRNA. Nuclease treatment of body-labeled Dsi (BL) produced a very similar pattern of bands (Figure 4E). The above experiment shows that RPA readily distinguishes 2–3 nt size differences and by comparing Dc9si and Tc9si (Figure 2) we conclude that there is no significant size difference between Dc9si and Tc9si.

**Figure 7:**
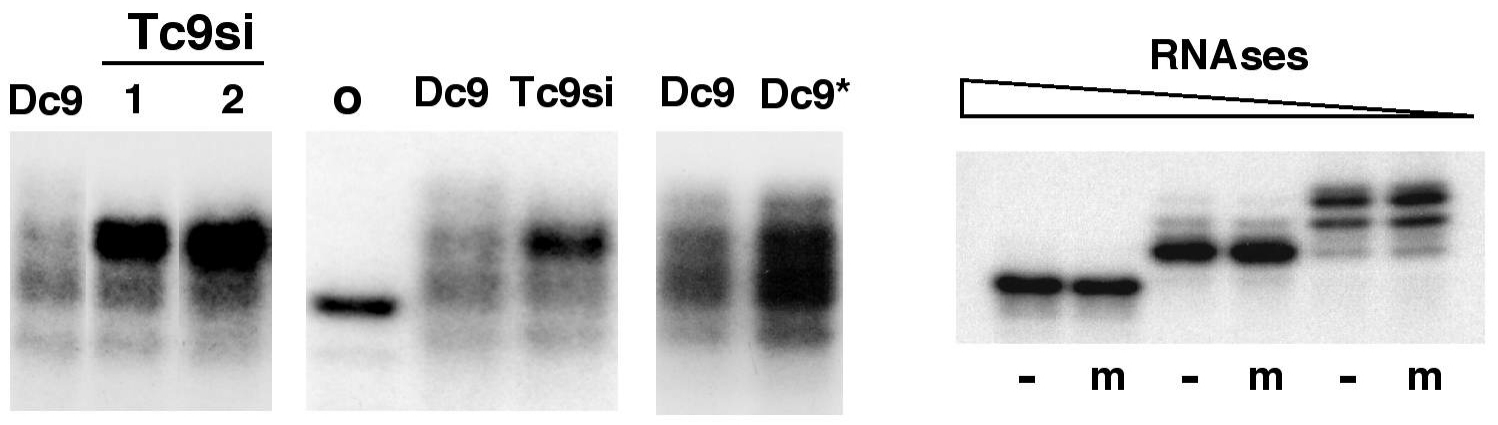
**Structure of plant-derived siRNAs**. Analysis of siRNA by RPA on non-denaturing gels. Native RPA products from single synthetic c9si (o), Dc9si and three different Tc9 total RNA samples were analysed on a 15% non-denaturing PAGE gel (left hand side). Dc9si^*^ that had been subjected to the Tc9 RNA extraction and purification regime (as described in Figure 4D) was also examined. Esi (-) or mEsi (m) (right hand side) were subjected to RPA analysis using increasing amounts of RNAses as indicated.

In denaturing urea gels the products observed following RPA are derived only from the ^32^P-labeled riboprobe. We reasoned that analysis on non-denaturing gels, in which the probe/plant siRNA heteroduplex would remain intact, might reveal conformational and hence structural differences between Dc9si and Tc9si. RPA was performed as usual except that samples were not heated and were resolved on a polyacrylamide gel under non-denaturing conditions (Figure 7). The gel pattern observed for Dc9si was multiple diffuse bands with no obvious bias, whereas in contrast, Tc9si exhibited a predominant slower migrating species that accounts for the major portion of the siRNA. A reproducible pattern was obtained for different Tc9si samples and for siRNAs from tomato (data not shown). To confirm that the altered Tc9si gel mobility was not caused during RNA preparation, we examined Dc9si^*^ siRNAs (as described in Figure 4) and found that the profile of bands for Dc9si^*^ on non-denaturing gels was not obviously altered (Figure 7). The above results suggest that the decreased gel mobility of probe/Tc9si hybrids in non-denaturing gels may reflect physiological modification of a substantial fraction of Tc9si. We note that the Dc9si sample contains a band of similar migration to the putative modified species from plants. While this might conceivably reflect an siRNA modifying activity of Dicer, it seems more likely that the Dicer-generated band simply comigrates with the putative modified plant siRNA. Thus we suggest that the putative modifying activity is plant-specific.

To assess whether a single 3'-2-O-methylation could account for the altered mobility of plant siRNAs in non-denaturing gels, we analysed Esi and mEsi siRNAs (Figure 7). Under a range of RNAse digestion conditions Esi- and mEsi-probe complexes showed no difference in migration relative to each other, indicating no effect of methylation on siRNA mobility under non-denaturing gel conditions. The above result indicates that 3'-2-O-methylation does not account for the altered gel mobility observed for T9si, suggesting that Tc9si may harbour a hitherto undetected siRNA modification.

## Discussion

### Transgenic plants as siRNA bioreactors

Compared with efficient gene silencing achieved by Dc9si, Tc9si show no activity in mammalian cells and we have performed a range of controls to show that the lack of activity is intrinsic to the siRNAs present in Tc9si. For technical reasons related to siRNA yield and purity we were unable to test concentrations of Tc9si (100 nM) that are often used for gene silencing experiments. It therefore remains possible, or even likely, that the Tc9si population is not totally inactive but rather, contains a mixture of species (see below) that exhibit low overall silencing activity. In any event potent siRNAs are effective at 1 nM [37] and use of siRNA concentrations higher than 25 nM may elicit significant off-target effects [37,44] and thus we have shown that siRNAs present in the Tc9si sample are inoperative for gene silencing in mammalian cells under stringent conditions. Complexity of the Tc9si RNA produced from the ~400 bp dsRNA precursor is probably quite high (see below) and simpler siRNA pools (from 100–150 bp precursors) exhibit similar silencing activity [45]. Thus the difference in silencing activity between the Tc9 and Dc9 siRNA pools seems unlikely to be accounted for by the particular siRNA sequences present.

Our conclusions are in striking contrast to those of a previous report [28] which claimed that plant-derived siRNAs can provide a sustainable source of siRNAs for use in mammalian cells/organisms. However, consistent with our findings, the efficacy of plant-derived siRNAs previously reported appeared to be poor [28] when compared with effective positive control siRNAs [46]. Thus it is apparent that transgenic plants offer no obvious advantages over established methodologies for siRNA production.

At the outset of our study, short term use of siRNA pools for gene silencing was attractive because it eliminates the need to screen for effective single siRNAs. However advances in RNAi methodology now indicate that siRNA pools have significant limitations. One concern that the bigger the pool size the higher the chance of off-target effects in mammalian cells [37,44,47,48]. Secondly, longer (~27 bp) synthetic siRNAs (which are not produced in planta) are extremely potent [49] apparently because coupling to Dicer allows more efficient RISC formation [49,50]. In summary the best approach for large scale RNAi application now appears to be identification of potent, elongated synthetic siRNAs and not the use of siRNA pools.

### Structure of siRNAs derived from plant transgenes

There are different size classes of plant siRNAs [30,51,52] which might have differential function in mammalian cells. However, the size distribution of siRNAs for the Tc9si and Dc9si samples is not obviously different, indicating that siRNA size does not account for the lack of Tc9si function in mammalian cells. The broad siRNA size range within the Tc9si sample is probably explained as follows. We have used a 400 nt hairpin dsRNA that might, minimally, be expected to yield 20–40 different siRNAs. However there is microheterogeneity for processing of long dsRNAs [45] and multiple Dicers (DCL2, DCL3 and DCL4) are able to produce siRNAs from hairpin-transgenes in planta [53]. Thus the sequence complexity of the Tc9si population may be quite high.

Asymmetric incorporation of siRNA strands into RISC [10,22] and data from siRNA cloning [27] suggest that siRNAs extracted from plants might contain significant amounts of single-stranded siRNAs. In addition multiple plant Dicers produce overlapping siRNAs from hairpin transgenes [53] suggesting that Tc9si will contain a portion of aberrant (non-functional) siRNA duplexes derived from distinct parents. However if stringent asymmetric RISC assembly applies then for any given pair of overlapping siRNAs, complementary strands will occur only 50% of the time and thus extracted siRNAs would be expected to contain significant amounts of single stranded siRNAs. Because our direct analysis of the Tc9si sample (Figure 5) did not detect appreciable amounts of single-stranded siRNAs, we therefore interpret this to indicate that a significant portion of the Tc9si RNAs are bona fide double-stranded siRNAs. This interpretation is consistent with genome scale smRNA sequencing analysis [54] suggesting that plants do not strictly follow the siRNA asymmetry rules. Considering all of the above it is most likely that the Tc9si sample harbors a mixture of aberrant and normal siRNAs.

Several factors could account for accumulation of a certain level of double-stranded siRNAs in the Tc9si sample. Firstly, similar to mammalian RISC [37] plant RISC is likely to be readily saturated. Second, siRNA production from CMV 35S-driven hairpin transgenes is very high (for example > 50-fold higher than that driven by a moderately active endogenous promoter [55]) thus saturating RISC and leading to accumulation of double-stranded siRNAs. In support of this suggestion, hairpin transgene processing operates through the viral defence pathway [53] and siRNAs extracted from virally-infected plants are, at least in some cases, double-stranded [56].

Recently it has been shown that, similar to plant miRNAs [57], all classes of siRNAs in *Arabidopsis *undergo HEN1-dependent 3' terminal 2-O methylation [39] which protects against nucleolytic degradation. Similarly a large fraction (~90%) of tobacco siRNAs have a 2-O modification (presumably methylation) of the 3' terminal ribose [40]. Our results (Figure 7) provide indirect evidence for an siRNA modification in addition to methylation, at least for siRNAs produced from plant transgenes. Proof of the putative plant-specific modification will depend on direct structural analysis.

### Function of plant siRNAs in mammalian cells

The previous finding that modified siRNAs can act as efficient stoichiometric RNAi inhibitors [37] and that Tc9si do not inhibit silencing by Dc9si (our study) suggests that Tc9si may not compete for RISC formation. This may indicate that Tc9si do not efficiently enter the mammalian gene silencing pathway. Ago2 (the mammalian slicer) interacts with the siRNA 3' overhang [58,59] and siRNA 3' 2-O methylation reduces Ago2/siRNA interaction [58]. However we have shown that (under stringent conditions) 3' 2-O siRNA methylation is not inhibitory to mammalian gene silencing and thus the effect of methylation on Ago2/siRNA interaction does not appear to compromise plant siRNA function in mammalian cells.

The ineffectiveness of Tc9si in mammalian cells might be explained by the presence of a significant proportion of aberrant siRNA duplexes (see above) together with the fact that we were unable to assay high concentrations of Tc9si (for the reasons mentioned above). It is also possible that the putative plant siRNA modification that we have proposed might reduce Tc9si activity in mammalian cells. In light of the complexity and diversity of plant RNAi signaling [7,60,61], involving multiple DCL (DCL1-4) and Ago family members [62], it is reasonable to suggest that siRNAs may contain structural features (other than size) that could directly contribute to specialisation. In relation to the above possibility, it would be of interest to examine whether specific plant DCL or Ago proteins [63] might be able to bridge the apparent gap between mammalian gene silencing components and plant siRNAs. Such experiments might provide important insights into conservation or divergence of the RNAi machinery in different organisms.

## Methods

### Plasmids

Plasmid pBmC9 (Figure 1A) for creation of transgenic plants and expression of siRNA pools from an ~400 bp long hairpin precursor was designed as previously described [28,29]. pBmC9s is related to pBmC9 but lacks the TGA1 intron [64] and mC9 antisense sequence and therefore expresses an 400 bp mC9 sense transcript. pEYFP-T2m expresses the EYFP protein in plants [65]. pBSmC9 contains the entire mouse caspase 9 cDNA sequence inserted into the polylinker of pBluescript SKII(-). pmC9-dsEGFP expresses a caspase 9/dsEGFP fusion protein containing the n-terminal 100 residues of mC9 fused to EGFP and was obtained as follows. pdsEGFP expresses dsEGFP and was derived from pmC9-dsEGFP.

### Generation and characterisation of transgenic tobacco

pBmC9 was introduced into tobacco (cultivar Samsun NN) via standard Agrobacterium-mediated transformation as described [28] using Agrobacterium strain GV3101/MP90 [66] and the leaf-disc procedure [67]. Primary transformants were first screened for GUS expression by leaf staining and transgene insertion was confirmed by PCR analysis of genomic DNA. Selected transformants were then tested for siRNA expression by Northern blotting (Figure 1).

### Preparation of plant total RNA and gene silencing in planta

RNA was originally prepared using TRIzol reagent (Invitrogen) according to the suppliers protocol except that much less TRIzol was used. Since TRIzol is the major cost factor for plant siRNA preparation we optimised the process and found that for a fixed volume of TRIzol, siRNA yield was proportional to the amount of leaf material below ~4 g of leaf/ml TRIzol. Accordingly a ratio of 1 ml TRIzol per 4 g leaf material (much less TRIzol than the suppliers recommendation) was used for larger scale siRNA extractions. For transient gene silencing assays in tobacco, leaves were infiltrated by injection of transformed Agrobacteria as previously described [28], total RNA was prepared 48 hours later and Northern blotting was performed using 20 μg of RNA.

### siRNA detection

Northern blotting [68] and RNase Protection Assays (RPA) [69] were carried out essentially as described elsewhere. For RPA a ^32^P-labeled anti-sense probe spanning ~200 nucleotides of the mC9 RNA sequence expressed in transgenic plants were prepared from linearised pBSmC9 and gel purified. Hybridisations contained 2 μl of RNA sample, 1 μl RNA probe (approximately 1% of the material obtained from in vitro transcription of 1 μg of a pBSmC9), 3 μl of 10× buffer (4 M NaCl, 400 mM Pipes pH 6.4, l0 mM EDTA) and deionized formamide in a total volume of 30 μl. Samples were denatured at 85°C for 10 minutes and then incubated at 45°C overnight. The mixture was treated at 30°C for 20 minutes with 20 μg/ml RNase A and 1 μg/ml RNase T1 following addition of 350 μl digestion buffer (l0 mM Tris pH 7.5, 5 mM EDTA and 300 mM NaCl). Reactions were stopped with SDS and proteinase K treatment, extracted with phenol/chloroform and the nucleic acid precipitated with ethanol after addition of 15 μg tRNA. Samples were then analysed on denaturing polyacrylamide gels. For non-denaturing gel analysis, after RNase protection, samples were loaded directly onto the gel by addition of 6× gel-loading buffer containing bromophenol blue, xylene cyanol and 30% glycerol.

### siRNA purification and quantitation

Small plant RNAs (TsRNAs) and DsiRNAs (Dsi) were purified from 15% non denaturing polyacrylamide gel. RNAs co-migrating with Dsi as were eluted in 350 μl depc-treated water at 37°C overnight, recovered by ethanol precipitation and resuspended in depc-treated water. The amount of Dsi was determined by ethidium bromide staining of 15% non denaturing polyacrylamide gels and by comparison with known amounts of synthetic siRNAs. Specific plant siRNAs (Tc9si) were then estimated by RPA and comparison with Dsi. For polysaccharide removal, siRNA samples were adjusted to 200 mM potassium acetate, incubated on ice for 30 minutes and then centrifuged for 30 minutes at 12,000 g in an Eppendorf microcentrifuge in the cold room. A clear pellet was observed and the supernatant (containing the siRNA) carefully removed. Polyvinylpyrrolidone (PVP) treatment was performed by including 2% water soluble PVP (M. Wt. 40,000; Sigma) during nucleic acid/lipid complex formation at the time of transfection.

### Nuclease and serum treatment of siRNAs

For limited RNase digestion, 3 ng of Dc9si RNAs (Dc9si) was incubated with 2 μg/ml RNase A and 0.1 μg/ml RNase T1 in 15 μl for 15 minutes at room temperature. The reaction was stopped by vortexing with 15 μl Phenol/Chloroform and 2 μl of the aqueous phase was analysed by RPA. Serum treatment of siRNAs was carried out by incubating 3 μl (4.5 ng) of each of Tc9si and Dc9si with 5 μl of FBS at 37°C. Reactions were stopped by adding 10 μl of REB (100 mM tris pH7.5, 150 mM NaCl, 12.5 mM EDTA, 1% SDS), followed by Phenol/Chloroform extraction and ethanol precipitation. The RNA pellet was dissolved in 10 μl depc-treated water and 2 μl (~0.5 ng siRNA) and analysed by RPA.

### Synthetic siRNAs

The sequences of synthetic mouse caspase 9 (c9si) and EGFP (Esi) siRNAs (Proligo) are as follows. Caspase 9 (c9si, sense, 5'-ggagcagagaguagugaagtt-3'; antisense, 5'-cuucacuacucucugcucctt-3') and EGFP (Esi, sense, 5'-gcaagcugacccugaaguucau-3'; anti-sense, 5'-gaacuucagggucagcuugccg-3'). mEsi is identical to Esi except that the 3' terminal ribonucleotide on both strands is methylated at the 2 position of the ribose. Dc9si were processed from ~400 nt double-stranded RNA precursors in 15 μl reactions under optimised conditions that were similar to the suppliers protocol (Stratagene). Using ~200 ng of dsRNA, 1 U of recombinant Dicer and incubation at 37°C overnight ~80% of the dsRNA was converted to siRNAs using an optimal amount of Dicer.

### Gene silencing assays in mammalian cells

BalbC 3T3 cells in 96-well tissue culture plates were co-transfected with pmC9-dsEGFP and pdsEGFP (70 ng each) and test siRNAs samples using Lipofectamine 2000 (Lipo2000, Invitrogen) according to the suppliers procedure. 16 hours after transfection cells from one transfected well plus one untransfected well (included as carrier to increase the cell pellet) were washed with PBS, treated with trypsin at 37°C for 5 minutes, neutralized by addition of growth medium containing 10% FBS, collected by centrifugation and washed again with PBS. Cells were then lysed by addition of 8 μl of cell lysis buffer (20 mM Tris-Cl, pH 7.5); 50 mM NaCl; 1 mM EDTA, pH 8; 1 mM EGTA, pH 7; 1% Triton X-100 and supplemented with complete protease inhibitor cocktail (Roche), centrifuged at 13000 g for 10 minutes and half of the cell lysate analysed by Western blot. Membranes were incubated for 1 hour with 1:2000 primary EGFP antibody (mouse monoclonal JL8, Clontech) and for 30 minutes with anti-mouse HRP conjugated secondary antibody (Amersham NA931). Visualisation was achieved using an ECL detection kit (Amersham NA931) with typical x-ray film exposure times of less than one minute.

## Authors' contributions

Bess Chau performed experimental work, designed methodology and contributed to writing the manuscript. Kevin Lee developed methodology and wrote the manuscript.
